# A blind spot in mental healthcare? Psychotherapists lack education and expertise for the support of adults on the autism spectrum

**DOI:** 10.1177/13623613211057973

**Published:** 2021-11-26

**Authors:** Silke Lipinski, Katharina Boegl, Elisabeth S Blanke, Ulrike Suenkel, Isabel Dziobek

**Affiliations:** 1 Humboldt-Universität zu Berlin, Germany; 2 Friedrich-Schiller-Universität, Germany; 3 University Hospital of Tübingen, Germany; 4 Charité – Universitätsmedizin Berlin, Germany; 5 Freie Universität Berlin, Germany; 6 Berlin Institute of Health, Germany

**Keywords:** autism spectrum condition, mental health, participatory research, psychotherapy, support, therapy, treatment

## Abstract

Most adults on the autism spectrum have co-occurring mental health conditions, creating a high demand for mental health services – including psychotherapy – in autistic adults. However, autistic adults have difficulties accessing mental health services. The most-reported barriers to accessing treatment are therapists’ lack of knowledge and expertise surrounding autism, as well as unwillingness to treat autistic individuals. This study was conducted by a participatory autism research group and examined 498 adult-patient psychotherapists on knowledge about autism and self-perceived competency to diagnose and treat autistic patients without intellectual disability compared to patients with other diagnoses. Psychotherapists rated their education about autism in formal training, and competency in the diagnosis and treatment of patients with autism, lowest compared to patients with all other diagnoses surveyed in the study, including those with comparable prevalence rates. Many therapists had misconceptions and outdated beliefs about autism. Few had completed additional training on autism, but the majority were interested in receiving it. Greater knowledge about autism was positively linked to openness to accept autistic patients. The results point to an alarming gap in knowledge necessary for adequate mental health care for individuals with autism.

**Lay abstract:**

Most autistic adults experience mental health problems. There is a great demand for psychotherapeutic support that addresses the specific needs of autistic individuals. However, people with autism encounter difficulties trying to access diagnostic and therapeutic services. This study was conducted by a participatory autism research group: a group in which autistic individuals and scientists collaborate. The group developed a questionnaire for psychotherapists in Germany to assess their knowledge about autism. Psychotherapists also rated their ability to diagnose and treat autistic patients without intellectual disability, and patients with other psychological diagnoses. Many of the 498 psychotherapists that responded reported little knowledge and outdated beliefs about autism, as well as little training on treating patients with autism. Their expertise about other psychological conditions was more comprehensive. However, many psychotherapists were interested in professional training on autism. Those with more knowledge were also more open to treating autistic patients. In conclusion, psychotherapists’ lack of knowledge and expertise seem to be a major barrier for adults with autism to receiving helpful psychotherapeutic support. The results demonstrate the need for an advancement in autism education during psychotherapists’ training and in continuous education.

Autism spectrum disorder (ASD) (hereafter autism spectrum condition, ASC)^1^ is characterized by difficulties in social communication and differences in behaviour, and sensory peculiarities (American Psychiatric Association, 2013) with a prevalence of approximately 1% (Brugha et al., 2011). However, intellectual variability in the autism spectrum is high, with 46% in the average or above average range of intellectual ability (IQ >85) (Centers for Disease Control and Prevention, 2020). The focus of this study is on those adults with ASC without intellectual disability.

Most autistic individuals suffer from some form of co-occurring mental health condition throughout their lifetime (Lever & Geurts, 2016), which can cause substantial impairment in adaptive functioning and in quality of life (Farley et al., 2009). At present, more than half of autistic adults are burdened with mental health problems (Hofvander et al., 2009), such as anxiety disorders (27%), obsessive-compulsive disorder (OCD; 24%), depression (23%) (Hollocks et al., 2019) and/or the co-occurrence of neurodevelopmental disorders such as attention-deficit/hyperactivity disorder (ADHD; 28%) (Lai et al., 2019). Some conditions may still be under-diagnosed given the unique presentation of mental health difficulties and the lack of validated assessment tools able to accurately detect comorbid psychological conditions in ASC (Cassidy et al., 2018a, 2018b) Mental health conditions contribute to the alarmingly high rates of suicidality in autistic adults (Hedley & Uljarević, 2018). Furthermore, a lack of support for this group has been associated with an increased risk of depression and suicidality (Cassidy et al., 2018c).

Hence, autistic adults need mental health support, most of whom seek it out (74% Gawronski et al., 2011; 73% Baldwin & Costley, 2015). Previous research suggests that autistic adults benefit from psychotherapy for co-occurring psychiatric conditions (Murphy et al., 2018; Russell et al., 2013, 2020), especially from cognitive behavioural therapy (CBT; Spain et al., 2015). However, compared to the general population, the number of unmet mental health needs for autistic adults is higher (Cassidy et al., 2018b).

In many countries, autistic adults must reach out to unspecialized psychiatrists and psychotherapists (Raja, 2014), as there are few specialized autism outpatient clinics, which usually have long waiting lists (Davidson et al., 2015). Thus, autistic adults commonly report difficulties in accessing community mental health services and outpatient psychotherapy (e.g. Lipinski et al., 2019). If treatment takes place, service provision is seldomly tailored to their needs (National Institute for Health and Care Excellence [NICE], 2016), and autistic adults often do not receive quality, evidence-based mental health care (Maddox et al., 2019; Roux et al., 2015). Psychotherapists’ poor knowledge about autism may negatively affect treatment satisfaction (Lipinski et al., 2019). In line with this, a lack of therapists’ knowledge and expertise, as well as their unwillingness to tailor approaches to autistic adults was identified as the most commonly reported barriers to accessing psychological treatment (Adams & Young, 2020). Research that contributes to the improvement of mental health care is of top priority to the autism community (Pellicano et al., 2014).

In the field of mental healthcare for adults with ASC, there is a high demand for psychotherapeutic treatment of comorbid psychological diagnoses (Lipinski et al., 2019). However, psychotherapeutic services for autistic adults have attracted barely any attention in clinical psychology and psychiatry in Germany. Despite the suffering, attention to autistic adults’ psychotherapeutic needs is paid almost exclusively at only a few specialized outpatient clinics. In the United States, Maddox and colleagues (2019) interviewed 22 autistic adults with community mental healthcare experience, 44 community mental health clinicians and 11 mental health agency leaders (Maddox et al., 2019). All stakeholders reported that low levels of knowledge and experience of clinicians working with autistic adults were a problem.

In Germany, psychotherapeutic treatment in an outpatient setting is only administered in local psychotherapeutic practices by licensed psychotherapists. However, Lipinski and colleagues (2019) reported on barriers to accessing local psychotherapeutic services for autistic adults and identified predictors of treatment satisfaction in a quantitative study with 262 autistic adults compared to non-autistic individuals with depression in Germany (Lipinski et al., 2019). Specifically, from the point of view of autistic adults, low levels of expertise with autism was a main reason for being declined therapy, and for individuals who did receive therapy it contributed to lower treatment satisfaction.

Due to its large contribution to health outcomes and quality of life, we seek to further investigate the status of and reasons for the paucity of psychotherapeutic treatment for autistic adults by surveying standard care psychotherapists. This study seeks to quantitatively ascertain through a nationwide survey whether autistic adults’ reports on low levels of education in psychotherapists are consistent with German standard care psychotherapists’ self-reported levels of education and knowledge about autism – particularly in adults. In addition, this study aims to provide insight into therapists’ self-rated training and competency levels compared to other patient groups.

We expected that psychotherapists would have scarce knowledge of autism and results would reflect prior reports by autistic adults. *Limited knowledge of autism* would be reflected in less education on autism, less self-indicated diagnostic competency, less treatment competency for autism than for similarly prevalent disorders, like ADHD, borderline personality disorder (BPD) and eating disorders (EDs), and misconceptions about the nature and pathogenesis of autism as shown by a lower scoring on questions regarding objective knowledge of autism.

Furthermore, we assumed psychotherapists have little experience with diagnosing autism and treating autistic patients compared to patients with similarly prevalent diagnoses.

In addition, this study explored factors that influence psychotherapists’ *openness to treat adults on the spectrum*, including self-rated openness to treat autistic adults, reasons given by psychotherapists for preferring not to treat autistic patients, and knowledge and patient characteristics perceived as interfering with therapeutic work. We expected to find therapists’ awareness and demand for better knowledge of ASC reflected in psychotherapists’ level of interest in further education on this topic.

## Methods

### Procedures

The study involved a cross-sectional survey on psychotherapists’ treatment knowledge and experiences with patients with various psychological disorders (autism, ADHD, BPD, depression, ED, OCD, phobias and schizophrenia). All measures were completed as part of an online survey conducted using SoSci Survey 2.0 (Leiner, 2019) and took approximately 10 min to complete.

No a priori sample size was calculated as we aimed for a maximum number of participants. From August 2016 through January 2017, a convenience sample of psychotherapists was recruited through professional organizations of psychotherapists and psychotherapist training institutes throughout Germany. To accommodate unequal participation rates, further participants were randomly recruited in some federal states via email. We stopped data collection when no more responses were received.

Since therapists with a keen interest in autism might have been more likely to respond to such a survey, we did not disclose that the survey’s underlying target condition was autism (neither in the call for participation nor in the survey’s introductory and explanatory texts). To further ensure blindness to the target condition, participants were asked how they had learned about the survey. Participants who had been made aware through a person with ASC were excluded.

Participants were given the option to take part in a raffle for 10 shopping vouchers worth 25 Euros each and to receive the results of the study, as an incentive to take part. All participants remained anonymous.

### Participants

In total, 577 therapists started the survey with 518 fulfilling the cut-off criteria (at least two-thirds of the questions in the questionnaire had to be answered). We further excluded 20 participants due to other criteria (i.e. participants had to have at least 1 year of psychotherapeutic practice experience; see Supplemental material for exclusion details). The final sample comprised 498 participants from all German federal states. Specific data on socioeconomic status and race/ethnicity were not recorded.

There were significantly more females than males in the sample (75.1% female, *χ*^2^(1) = 125.5, *p* < 0.001). On average, the male participants were older than the female participants ((M_female_ = 42.8, standard deviation (SD)_female_ = 12.1; M_male_ = 52.6, SD_male_ = 13.2), *t*(196.10) = 7.34, *p* < 0.001). Accordingly, male psychotherapists reported more years of treatment experience ((M_female_ = 9.5, SD_female_ = 9.2; M_male_ = 17.6, SD_male_ = 12.3), *t*(171.72) = 6.79, *p* < 0.001).

Most participants (*N* = 363; 72.9%) were licensed psychotherapists, and 135 (27.1%) participants were still engaged in supervised training. In addition, 181 (36.3%) participants were working in a clinic or psychotherapist training institute’s outpatient clinic, 256 (51.4%) participants were working in a private psychotherapeutic practice, and 61 (12.2%) participants were working in both. Of the whole sample, 475 (95.4%) participants were psychological psychotherapists, and 23 (4.6%) participants were medical psychotherapists. Therapists needed to carry out at least one of the following three therapeutic approaches, given that these are approved and paid for by the German compulsory health insurance: CBT, psychodynamic psychotherapy or analytical psychotherapy. The majority (55.4%) of the psychotherapists were trained in CBT (*n*=276) or in psychodynamic psychotherapy or analytical psychotherapy (41%; *n*=204). 3.6% (*n*=18) were trained in both CBT and either psychodynamic psychotherapy or analytical psychotherapy. Since therapeutic method, age and gender were highly interrelated within our sample (e.g. relatively more males were trained in in-depth psychology-based treatment and males were significantly older than females), we refrained from conducting group comparisons between therapeutic methods. The average treatment experience of participants was 11.5 years (SD = 10.7) with a mean age of 45.2 years (SD = 13.0, range = 26–77).

The study was conducted in line with the principles of research ethics established in the Helsinki Declaration. Participants gave informed consent by entering the survey after the nature of the study and data protection procedures had been explained on the initial page.

### Measures

All participants completed questions about their sociodemographic background.

### Knowledge on autism

To investigate psychotherapists’ knowledge about autism, participants were asked about their experience with psychotherapeutic training and their self-perceived competency in the assessment and treatment of patients with autism, ADHD, BPD, depression, ED, OCD, phobias and schizophrenia on a scale from 0 (the lowest score) to 100 (the highest score). As no standardized questionnaires existed, items for this purpose were created by the participatory research group that conducted the study (see section ‘Community involvement’).

Due to the lack of a comparable and evaluated questionnaire surveying autism in adults, items to investigate general knowledge of autism were selected from a well-established autism survey developed by Stone (1987). The original questionnaire was comprised of 22 beliefs regarding social/emotional and cognitive aspects, its treatment/prognosis in children, and was based on common misconceptions about ASC derived from research and practice (e.g. ‘Most autistics are affected by intellectual disability’ and ‘Autistic withdrawal is partially due to cold, rejecting prior attachment figures’). Similar items were used in studies to evaluate knowledge and attitudes among health care workers and paediatricians in various countries (e.g. Effatpanah et al., 2019; Hayat et al., 2019; Rahbar et al., 2011). The participatory research group selected the 12 items applicable for knowledge about adult autism and rephrased them to be age neutral. Two additional items reflecting widespread belief patterns about autism were created (‘Autism can be caused by vaccination’ and ‘Autistics tend to be aggressive towards other people’; for all items, see Figure 2). Participants had to indicate whether they agreed, disagreed, or remained undecided about the statements’ content. We calculated an individual autism knowledge score – defined as the individual number of correct answers to the 14 questions – targeting general knowledge about autism (range = 0–14). Furthermore, participants were asked to quantify their experience with diagnosing autism and treatment of autistic patients compared to patients with similarly prevalent diagnoses.

### Openness to treat adults on the spectrum

To investigate psychotherapists’ *openness to treating adults on the spectrum*, participants were asked to indicate to what extent they can envisage treating an adult with autism on a Likert-type scale ranging from 1 (‘I can’t envisage treating a patient with autism’) to 4 (‘I can well envisage treating a patient with autism’). In addition, all participants were asked to give reasons why they would be reluctant or refuse to treat someone with autism. The answer format was multiple choice with an additional custom entry option. Items covered a range of possible concerns (e.g. ‘The application procedure for reimbursement is complicated’, ‘The otherness of autistic people seems strange/disconcerting to me’ and ‘I can’t perform psychotherapy without eye contact’; see Supplemental material for all items).

To investigate factors that might influence psychotherapists’ willingness to treat patients with autism, eight items were created on potential patient characteristics associated with ASC symptomatology. Importantly, in these items, ASC characteristics were highlighted without explicitly mentioning them as being part of an autism diagnosis, to identify barriers that are associated with behaviours associated with ASC, rather than the diagnostic label. The question was phrased as follows: ‘In your opinion, how much do restricted capacities of patients in the domains listed below interfere with psychotherapeutic treatment?’ Items were rated on a 4-point Likert-type scale ranging from 1 (‘Does not interfere with treatment’) to 4 (‘Extremely interferes with treatment’) and pertained to the following domains: interpersonal abilities/relating to others, non-verbal abilities (eye contact, mimics and gestures), impulse control, introspection, social interaction, oral articulateness/verbal skills, ability to change and awareness of own emotions (see Supplemental material for all items).

Finally, participants were asked if they had ever pursued/received continuing education (CE) on psychotherapy for autistic adults and if, generally, they would like to receive CE on this topic.

### Community involvement

This public health research project was carried out by the Autism Research Collaboration (Autismus-Forschungs-Kooperation; AFK, n.d.), a voluntary participatory research group comprised of mostly autistic adults and several scientists researching autism in Germany. Academic and autistic research partners have been meeting on a regular basis since 2007 and collaborate as research partners to develop and conduct research that is both important to the members on the autism spectrum as well as academically relevant. All members of the group aim to ensure that the research is inclusive, respectful and scientifically sound.

All decisions regarding the topic, design and execution of the study were made jointly and all parts of the study were conducted by the participatory research group, including data interpretation and decisions about the dissemination of study results (e.g. at scientific conferences). Both autistic and non-autistic participatory research group members authored this article, contributed to editing this article and wrote the plain language summary.

### Statistical analyses

Statistical analyses were performed using *R* (version 3.6.0, R Core Team, 2019). To determine sociodemographic characteristics and to test for group specifics, we performed Pearson’s chi-square tests and Student’s *t*-tests. Differences between diagnoses regarding self-perceived education, assessment competency and treatment competency were evaluated by Tukey’s tests.

To examine factors that influence openness to treating adults on the spectrum, we used the self-reported ‘openness to treatment’ as an outcome, and individual knowledge about autism and potential patient characteristics that impede conducting therapy as predictors. We focused on these two predictors because we assumed that they could be addressed and changed in prospective therapeutic training.

Preliminary analyses showed that only 3% of participants (*n* = 14) reported being unable to envisage treatment, which is why we split the 4-point scale into two categories with the levels 0 (‘cannot envisage’ and ‘would rather not envisage’) and 1 (‘can envisage with previous training’ and ‘can well envisage’) and used this dichotomous score (hereafter referred to as ‘openness to treatment’) as the dependent variable of a logistic regression. In a second preliminary data analysis step, we summarized the eight items targeting patients’ characteristics that create a barrier to conducting therapy using exploratory factor analysis (EFA) with oblique Geomin rotation. We used the Kaiser criterion (eigenvalues >1) to determine a sensible number of underlying factors and used Cronbach’s alpha to evaluate the scores’ internal consistencies. The EFA revealed that the items are best represented by two factors: (1) ‘social barriers’ (SB), consisting of four items about interpersonal abilities (e.g. eye contact) and (2) ‘barriers of affect control and ability to change’ (AB), comprised of three items regarding introspection and the ability to change. The four items with high loadings on SB and the three items with high loadings on AB were summed up to create composite scores (SB-score and AB-score). One item (‘verbal skills’) was not assigned to any score due to its low factor loadings on both factors (see supplemental material). Cronbach’s alpha for both scores indicated an acceptable internal consistency with *α* = 0.65 for the SB-score and *α* = 0.62 for the AB-score.

We conducted the logistic regression to predict openness to treatment through individual knowledge about autism and the two barrier scores twice, once with the whole sample and once with a subsample of therapists that had never treated patients with ASC before (*n* = 318) since past autism treatment might confound the knowledge about autism and openness to treatment.

## Results

Here, we report the results of our analyses for our two main research foci, namely, therapists’ knowledge on autism and openness to treating adults on the spectrum. The number of participants who responded to individual survey items varies slightly. We provide the exact number of responses in parentheses after the results.

### Knowledge on autism

#### Education and competencies

**Figure 1.**
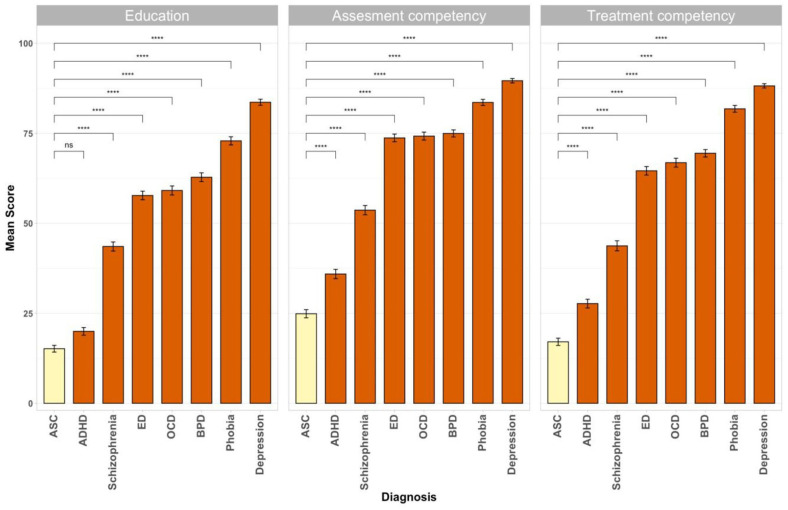
Mean values of self-evaluations with respect to different areas of therapeutic work. Participants rated their self-reported education, assessment competency and treatment competency for patients with different diagnoses on a scale from 0 to 100. Error bars represent the respective standard errors. Asterisks display the significant mean differences as computed by Tukey’s tests (*p* < 0.05). Answer rates varied between *n* = 497 (e.g. assessment competency OCD) and *n* = 445 (treatment competency ASC).

Mean values of self-reported education, assessment competency and treatment competency for patients with different diagnoses are displayed in Figure 1.

A large group of participants (53%) reported very little knowledge and/or autism-specific psychotherapeutic training, as indicated through a self-reported education score of <10, whereas only a few participants (2%) reported being highly educated (self-reported education score of >90).

#### Knowledge score

**Figure 2.**
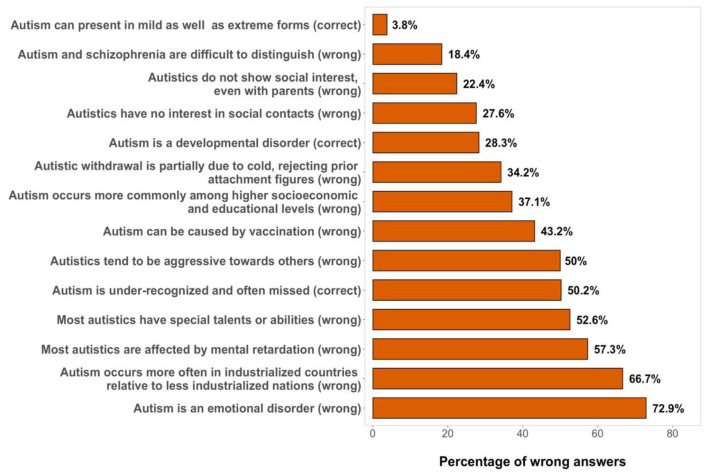
Percentages of incorrect answers to the 14 questions targeting general knowledge about autism. The *y*-axis labels correspond to the 14 questions targeting general knowledge about autism. The correct answer to each statement is shown in parentheses.

Out of 14 questions targeting general knowledge about autism, psychotherapists on average answered 8.3 (SD = 2.4) questions correctly. Percentages of incorrect answers and the correct option of each question are displayed in Figure 2. Not all participants answered these questions, but answer rates varied only between *n* = 421 and *n* = 425.

#### Experience

A subsample of 104 participants (21.1%, *N* = 493) had experience in diagnosing autism, whereas 376 participants had previously diagnosed BPD (76.0%, *N* = 495), and 332 had diagnosed ED (67.3%, *N* = 493). With respect to treatment, 130 participants indicated having carried out psychotherapy in individuals with autism (29.0%, *N* = 448), 414 in individuals with BPD (92.0%, *N* = 450) and 398 in individuals with ED (88.6%, *N* = 449).

### Openness to treating adults on the spectrum

Three-hundred and one participants (70.7%, *N* = 426) indicated that they were open to treating an autistic adult.

#### Reasons for not treating adults on the spectrum

**Figure 3.**
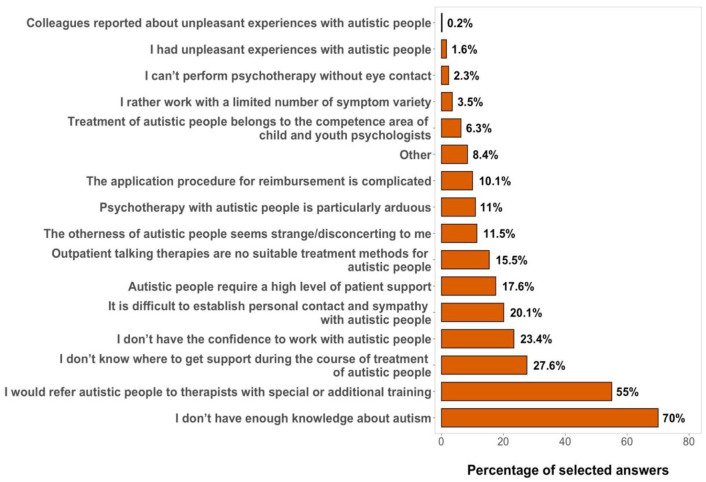
Possible reasons for not treating autistic patients sorted by relative selection. Percentages indicate how many therapists selected this answer. Multiple answering options could be chosen.

Possible reasons psychotherapists indicated for not treating, or being reluctant to treat, autistic patients (*N* = 427) are listed in Figure 3.

#### Factors influencing openness to treating adults on the spectrum

In combination with the individual knowledge score, we used the ‘social barrier’ score (SB-score) and the ‘barriers of affect control and ability to change’ score (AB-score) as predictors in a logistic regression with openness to treatment as the dependent variable. Higher individual knowledge about autism significantly predicted more openness to treatment (odds ratio (OR) = 1.2, *p* < 0.001). The second significant predictor in our regression was the SB-score (OR = 0.8, *p* = 0.001). Therapists that indicated experiencing larger interferences in conducting therapy when patients are restricted in social domains were less open to treating autistic patients. The last predictor, the AB-score, was not significantly related to openness to treatment (*p* = 0.62). To derive conclusions for therapists lacking experience with autism, we conducted the same analysis with the subsample of therapists that had never treated patients with ASC before (*n* = 318). Here, the only significant predictor was individual knowledge about autism (OR = 1.2, *p* < 0.01).

#### CE

**Figure 4.**
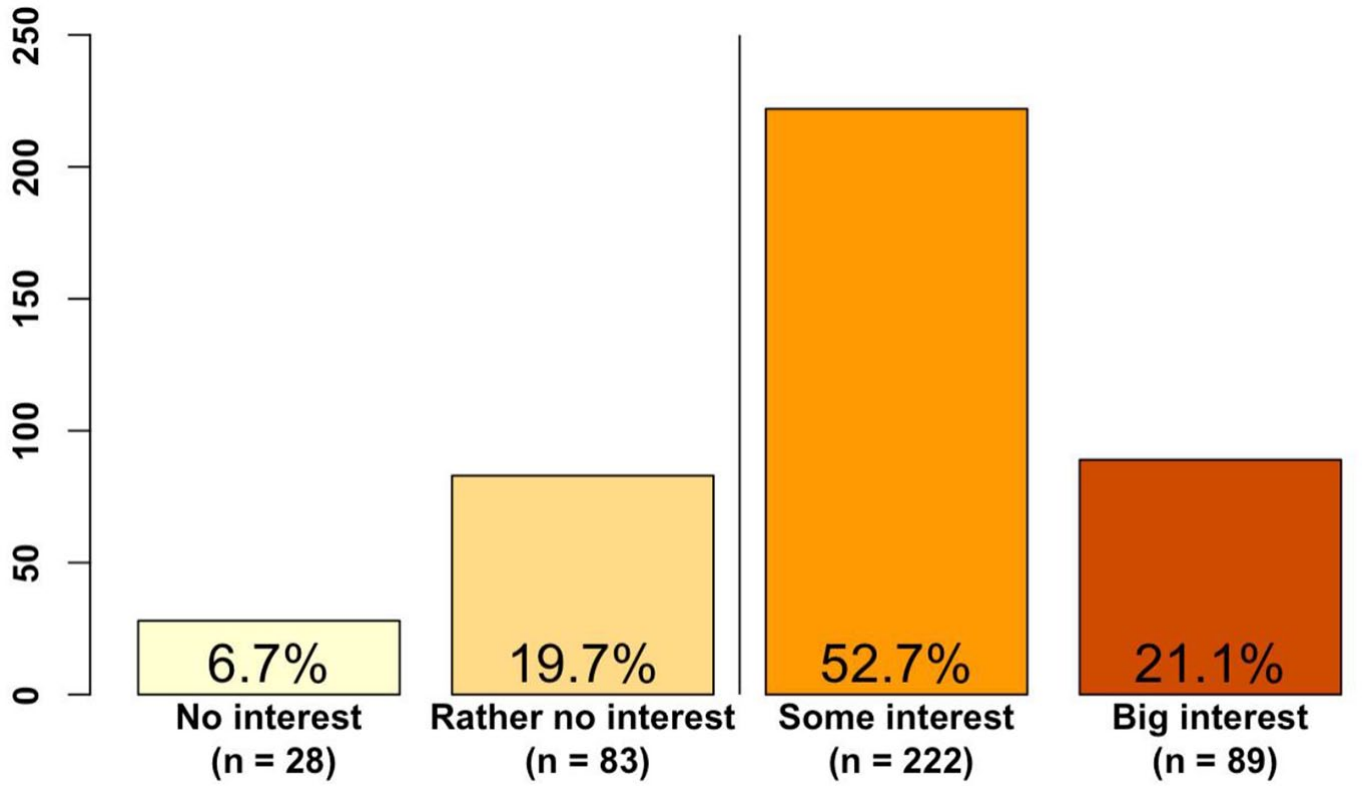
Participants’ interest in continuing education on psychotherapy for autistic adults. The *y*-axis represents the absolute numbers. Percentages are depicted inside the bars.

Sixty-three participants (14.9%, *N* = 422) reported having received CE on psychotherapy for autistic adults. Results on participants’ interest in CE in the future (*N* = 422) are displayed in Figure 4.

## Discussion

The overarching aim of this study was to get a picture of the current quality of psychotherapeutic services available to autistic individuals and to identify possible barriers to receiving mental health support. Regarding knowledge on autism, psychotherapists reported receiving significantly less education than all other diagnoses included in the study, with the exception of ADHD. In addition, many therapists had misconceptions and outdated beliefs about the symptoms and aetiology of autism. Psychotherapists self-evaluated their competency and experience in diagnosis and treatment of autistic patients lowest compared to that of patients with diagnoses of comparable prevalence rates.

We found that most therapists were open to treat individuals on the spectrum (with additional training) and were interested in receiving additional training. Hesitation to treat autistic patients was primarily credited to self-perceived lack of knowledge. Greater knowledge about autism and therapists’ ability to assist patients restricted in social domains was positively linked to openness to treating autistic patients.

### Limited knowledge on autism

Psychotherapists in our survey reported having received little education on ASC during formal training. Consequently, they had low experience and self-rated competency in treating clients with ASC. Psychotherapists also had misconceptions about ASC.

Participants overall reported having received significantly less education on autism during their formal training to become psychotherapists than BPD, depression, ED, OCD, phobias and schizophrenia. This demonstrates that education on autism is not routinely part of the curriculum in psychotherapeutic training institutes focusing on adult psychotherapy.

Only education about ADHD was rated about as low as autism. Like ASC, ADHD is still considered a condition related to childhood, and thus both ASC and ADHD may often not be incorporated into psychotherapy training curricula tailored to adults.

A review by Adams and Young (2020) identified therapists’ low level of expertise as the overall most common barrier to accessing mental health support, according to individuals with ASC. In 2019, our participatory research group surveyed a large group of autistic adults in Germany on their experiences accessing psychotherapeutic support compared to those of adults without ASC and depression. The most common reported reason for psychotherapy not coming about for adults with ASC was therapists’ low level of expertise on the topic, which mirrors the findings reported here. If psychotherapy was realized, after relationship quality, the most influential factor for satisfaction with treatment received was therapists’ knowledge about autism.

In line with the low levels of education, therapists’ self-perceived diagnostic and treatment competencies for autism were rated as being significantly less than all comparison diagnoses with comparable prevalence. The lack of diagnostic competency is worrying, given that therapists play an important role in the identification of previously undetected autism in patients seeking a therapist to treat comorbidities since the characteristics of autism can overlap with many indicators of mental health conditions (Stewart et al., 2006).

In addition, if psychotherapists lack competency in identifying when someone’s autistic symptoms are a significant factor, recognition of co-occurring mental health conditions is also impeded (Matson & Williams, 2013). This, again, is in line with autistic adults’ reports (Au-Yeung et al., 2019) about mental health professionals perceiving autism characteristics as symptoms of mental health conditions or, in turn, perceiving mental health difficulties as part of the autism symptomology.

Therapists’ ratings on competency in the treatment of autistic patients were significantly lower compared to patients with all other comparison diagnoses, which likely reflect the lack of education about autism. Psychotherapists who lack confidence in their ability to adequately support autistic patients may not accept adults with autism for treatment or may have problems adequately providing treatment. This interpretation is in line with reports of autistic adults struggling to find psychotherapeutic support due to therapists’ problems tailoring treatment to their needs (Adams & Young, 2020). Patients reported that the most frequent reason for being turned down is therapists’ lack of knowledge about/experience with autism, and that treatment would have been more helpful if the therapist had been more knowledgeable about their condition (e.g. Lipinski et al., 2019).

Low ratings in education on autism were also evident from the misconceptions many psychotherapists had about the nature and pathogenesis of autism. For example, more than a third of the participants of the survey held misconceptions of the role of vaccinations (43%) and cold, rejecting attachment figures (34%) as an etiologic factor for autism. Only two-thirds (63%) of psychotherapists knew that autism occurs across socioeconomic status (Volkmar et al., 1997), and only one-third (33%) were aware that autism prevalence is not connected to the level of industrialization of a nation. Twenty-two percent of our sample held misconceptions about a lack of social interest in autistic adults, when in fact, difficulties initiating and maintaining social contact at the desired qualitative/quantitative level is one of the major causes of limited quality of life (Schmidt et al., 2015) for which autistic patients seek help (Gawronski et al., 2011). Moreover, 57% of the psychotherapists thought that most autistic adults are affected by intellectual disability, which is not the case (46% of individuals with autism have normal or above average IQ; Centers for Disease Control and Prevention, 2020).

Misconceptions shape therapists’ perception and expectations of how an autistic patient might present. For example, therapists may incorrectly assume that patients with social connections or without intellectual disability are unlikely to be autistic. Anecdotal evidence by autistic adults supports this apprehension.

Overall, although psychotherapists have the resources and general expertise to care for individuals with autism and mental health issues, but they should have at least some basic knowledge about autism to deliver satisfactory treatment (e.g. Lipinski et al., 2019; Matson & Williams, 2013; Montazeri et al., 2020). Autistic patients depend on therapists’ knowledge about their syndrome more than patients from other diagnostic groups, not only to avoid frequently experiencing misunderstandings (Camm-Crosbie et al., 2019) but also to form positive therapeutic relationships (Woods et al., 2013). This is crucial, as the quality of the therapeutic alliance is an important predictor for the success of psychotherapeutic treatment overall (Horvath et al., 2011), but also in ASC (Lipinski et al., 2019). And yet, the results on knowledge about autism attest to psychotherapists having little knowledge and holding outdated beliefs.

Overall, knowledge about autism is poor, not only in psychotherapists but also in healthcare providers in general (e.g. Morris et al., 2019) and teachers (e.g. Vincent & Ralston, 2020). The misconceptions found in psychotherapists in this study were also found in the general population (Jones et al., 2021). Thus, education should be provided to the general public to reduce beliefs that contribute to stigmatization and to professionals for better health outcomes and quality of life for adults with ASC.

Participants’ experience in the assessment and treatment of patients with autism were few compared to BPD and EDs, despite the similar prevalence of the disorders. Given the low levels of education, knowledge and perceived competency, it becomes comprehensible that more than half of participants ‘would refer autistic people to therapists with special or additional training’. Thus, our respondents seem to suggest that treatment of this population requires specialized therapists, and that it does not fall within their scope of responsibility. Yet, there are too few specialist services (e.g. Murphy et al., 2018), resulting in autistic adults being underserved with respect to assessment and treatment.

### Openness to treating adults on the spectrum

Although most therapists (71%) were open to treating autistic adults, there were also concerns why therapists would rather not treat autistic adults (e.g. ‘Outpatient talking therapies are suitable treatment for autistic people’ (16%) and ‘I don’t have the confidence to work with autistic people’ (23%)). Most of these reasons may be addressed through education and training. Noticeably, a lack of knowledge is the primary reason for a reluctance to treat autistic patients.

Therefore, based on psychotherapists’ self-report and scientific evidence, substantial shortcomings exist in basic knowledge of autism in adult patients. Hence, it is not just a perception on the part of autistic adults that their access to treatment depends largely on the knowledge of the therapists (e.g. Lipinski et al., 2019). These results make a strong case for providing more education and training on autism to increase the number of psychotherapists taking on autistic clients.

In addition, 27% of therapists indicated not knowing where they could get support, suggesting that there is a shortage of knowledge on the level of supervision and materials available for psychotherapists. In fact, there is only one treatment manual for individual talk-based psychotherapy available in Germany (Dziobek & Stoll, 2019) and very few internationally (e.g. Gaus, 2019).

We further corroborated our finding that a lack of knowledge is a major reason for the lack of treatment willingness. Using an objective knowledge score, we found that the higher the knowledge score, the higher the chance that therapists would indicate being willing to treat autistic patients. The second significant predictor for the treatment willingness was a ‘social barrier’ score, which was comprised of ratings of the assumed impact of patients’ social impairments (such as eye contact) on therapeutic work. Therapists with a high ‘social barrier’ score were less open to treating autistic adults, for whom they likely infer such impairment.

When we examined the subgroup of therapists who have not worked with autistic individuals yet, the SB-score no longer significantly predicted openness to treatment. We speculate that therapists with experience with autistic patients might have a deeper understanding of the impact that social difficulties (like those in people with ASC) can have on the therapeutic process (e.g. building of therapeutic alliance). Therefore, their willingness to continue treating autistic patients might not only rest on their knowledge of ASC but also on their experience. This, again, may indicate insufficient prior training in working with such restrictions or not being able to find enough support during treatment (e.g. supervision competent in ASC).

In contrast to these effects, concerns about affect control and ability to change, as operationalized through the ‘concerns about affect control and ability to change score’, were not significantly related to openness to treatment. This supports our interpretation that it is mostly a lack of knowledge and concern about social competencies, and not general restrictions in autism that prevent therapists from treating autistic patients.

Thus, increasing knowledge about autism may lead to a higher openness to treating autistic patients. This result has strong implications for education programmes and universities, as places for the advancement of knowledge about autism among psychology students and future psychotherapists. For already licensed therapists, recommended improvements include easy access to CE and sufficient availability of supervisors competent in ASC.

Furthermore, most of our respondents (74%) indicated having an interest in receiving CE on ASC. However, only 15% of the respondents had already participated in CE on ASC. Therefore, the lack of education during training is not compensated through CE measures.

### Limitations

Since the survey was voluntary, it can be assumed that motivated therapists participated, and accordingly, generalization of the results might be difficult. However, in case of an overrepresentation of motivated participants, we expect the results to be positively skewed and the actual situation to be even worse. Future studies might also include more information about the treatment (e.g. frequency) and the therapists (e.g. neurodiversity of therapists).

The nationwide sample was comprised of more females (75%) than males; especially among participants with less than 10 years of treatment experience. The gender ratio reflects the increasing proportion of women (approximately 75%) in psychology degree programmes in Germany (Statistisches Bundesamt, 2020). In addition, more generally, an overrepresentation of female participants is common in online surveys (Rhodes et al., 2003; Sax et al., 2003).

Findings rely predominantly on therapists’ self-reports as there is no standard on how to assess health care providers’ knowledge regarding autism. We note that reliability and validity of the knowledge measure used in this study can be inferred only to a limited degree due to necessary adaptations to the original knowledge questionnaire by Stone (1987). The field of ASD knowledge assessment would benefit from the establishment of a measure for the subdomain of knowledge about autism in adults.

Finally, this survey was conducted in Germany only. Therefore, generalization of the results to other countries is limited. However, the findings are in line with previous research on knowledge about autism among members of medical and mental health care facilities in several countries (e.g. Camm-Crosbie et al., 2019). Consistently, low knowledge levels of providers were reported by autistic adults and identified as a major barrier for accessing mental health support in several countries. Hence, we assume similar mechanisms to be involved concerning barriers to treating autistic adults.

## Conclusion

Individuals with ASC and no intellectual disability belong to a patient population in desperate need of mental health support. So far, knowledge about autism, its diagnosis and how to tailor treatment to individuals with ASC seem to be a blind spot in psychotherapists’ education. The lack of education and knowledge plays a major role in therapists’ willingness to accept autistic adults for treatment. Thus, we suggest incorporating education about ASC into the standard training curriculum and making sure enough CE opportunities on autism in adults are offered to psychotherapists, to reduce the systematic blind spot in mental health support for individuals with ASC.

## Supplemental Material

sj-docx-1-aut-10.1177_13623613211057973 – Supplemental material for A blind spot in mental healthcare? Psychotherapists lack education and expertise for the support of adults on the autism spectrum Click here for additional data file. Supplemental material, sj-docx-1-aut-10.1177_13623613211057973 for A blind spot in mental healthcare? Psychotherapists lack education and expertise for the support of adults on the autism spectrum by Silke Lipinski, Katharina Boegl, Elisabeth S Blanke, Ulrike Suenkel and Isabel Dziobek in Autism

## Notes

1. We use the term ‘autism spectrum condition (ASC)’ rather than ASD, to be less stigmatizing and to convey that people on the autism spectrum show differences that include strengths as well as difficulties. This procedure is in accordance with recent research (Kenny et al., 2016) and the views of the participatory research group that conducted this study.
