# Supplementary material for: A blind spot in mental healthcare? Psychotherapists lack education and expertise for the support of adults on the autism spectrum
Source: Autism. 2021 Nov 26;26(6):1509–21. doi: 10.1177/13623613211057973 (PMC9344568; doi:10.1177/13623613211057973)
Supplement: sj-docx-1-aut-10.1177_13623613211057973 – Supplemental material for A blind spot in mental healthcare? Psychotherapists lack education and expertise for the support of adults on the autism spectrum [file sj-docx-1-aut-10.1177_13623613211057973.docx]

**Supplemental material**

**Exclusion criteria for participants:**

- Less than one year of work experience (9 participants)
- Indicated to training in all of the 3 therapeutic schools CBT & psychodynamic psychotherapy & analytical psychotherapy* (4 participants)
- Indicated to training in none of the 3 therapeutic schools CBT & psychodynamic psychotherapy & analytical psychotherapy (4 participants)
- Recruited through autistic patients (2 participants)
- Implausible values (1 participant)

* we excluded these participants prior to analyses because we originally planned to examine differences in self reports between therapeutic schools. However, as stated in the manuscript, therapeutic schools, age, and gender were highly interrelated which is why we refrained from further analyses.

**Items on potential patient characteristics which are associated with ASC symptomatology and that might be considered an impedement to conducting therapy**

In your opinion, how much do restricted capacities of patients in the domains listed below interfere with psychotherapeutic treatment?

Interpersonal abilities/relating to others

Non-verbal abilities

Impulse control

Introspection

Social interaction

Verbal skills

Ability to change

Awareness of own emotions

**Items on possible concerns to be hesitant or reluctant to treat a patient with ASC**

Outpatient talking therapies are no suitable treatment methods for autistic people.

Autistic people require a high level of patient support.

The application procedure for reimbursement is complicated.

The otherness of autistic people seems strange/disconcerting to me.

Treatment of autistic people belongs to the competence area of child and youth psychologists.

It is difficult to establish personal contact and sympathy with autistic people.

I rather work with a limited number of symptom variety.

I had unpleasant experiences with autistic people.

I don’t have the confidence to work with autistic people.

I don’t have enough knowledge about autism.

I would refer autistic people to therapists with special or additional training.

I don’t know where to get support during the course of treatment of autistic people.

Colleagues reported about unpleasant experiences with autistic people.

I can’t perform psychotherapy without eye contact.

Psychotherapy with autistic people is particularly arduous.

Other

**Factor loadings after geomin rotation**

| Loadings | F1 | F2 | Assigned |
| --- | --- | --- | --- |
| Interpersonal abilities/relating to others | .593 |  | SB |
| Non-verbal abilities | .609 |  | SB |
| Impulse control | .524 |  | SB |
| Introspection | .184 | .580 | AB |
| Social interaction | .683 |  | SB |
| Verbal skills | .335 | .220 | - |
| Ability to change |  | .682 | AB |
| Awareness of own emotions | .321 | .459 | AB |

Note. The item “verbal skills” was not incorporated into further analyses due to its low factor loadings. SB = “social barriers” score; AB = “barriers of affect control and ability to change” score
